# Impact of inpatient Care in Emergency Department on outcomes: a quasi-experimental cohort study

**DOI:** 10.1186/s12913-017-2491-x

**Published:** 2017-08-14

**Authors:** Aisha Lateef, Soo Hoon Lee, Dale Andrew Fisher, Wei-Ping Goh, Hui Fen Han, Uma Chandra Segara, Tiong Beng Sim, Malcolm Mahadehvan, Khean Teik Goh, Noel Cheah, Aymeric YT Lim, Phillip H. Phan, Reshma A Merchant

**Affiliations:** 1 University Medicine Cluster, National University Hospital, 1E Kent Ridge Road, Singapore, 119074 Singapore; 2 Yong Loo Lin School of Medicine, National University of Singapore, 12 Science Drive 2, Singapore, 117549 Singapore; 3 The Johns Hopkins Carey Business School, 100 International Drive, Baltimore, MD 21202 USA; 4 Strome College of Business, Old Dominion University, 2027 Constant Hall, Norfolk, VA 23529 USA; 5 Emergency Medicine Department, National University Hospital, 1E Kent Ridge Road, Singapore, 119074 Singapore; 6 National University Hospital, 1E Kent Ridge Road, Singapore, 119074 Singapore; 7 Department of Hand and Reconstructive Microsurgery, National University Hospital, 1E Kent Ridge Road, Singapore, 119074 Singapore; 8 Carey Business School & Department of Medicine, Johns Hopkins University, 100 International Drive, Baltimore, MD 21202 USA

**Keywords:** Emergency department, Boarders, Bed occupancy rates, Inpatient care, Length of stay, Bed allocation, Readmissions

## Abstract

**Background:**

Hospitals around the world are faced with the issue of boarders in emergency department (ED), patients marked for admission but with no available inpatient bed. Boarder status is known to be associated with delayed inpatient care and suboptimal outcomes. A new care delivery system was developed in our institution where boarders received full inpatient care from a designated medical team, acute medical team (AMT), while still residing at ED. The current study examines the impact of this AMT intervention on patient outcomes.

**Methods:**

We conducted a retrospective quasi-experimental cohort study to analyze outcomes between the AMT intervention and conventional care in a 1250-bed acute care tertiary academic hospital in Singapore. Study participants included patients who received care from the AMT, a matched cohort of patients admitted directly to inpatient wards (non-AMT) and a sample of patients prior to the intervention (pre-AMT group). Primary outcomes were length of hospital stay (LOS), early discharges (within 24 h) and bed placement. Secondary outcomes included unplanned readmissions within 3 months, and patient’s bill size. χ2- and Mann-Whitney U tests were used to test for differences between the cohorts on dichotomous and continuous variables respectively.

**Results:**

The sample comprised of 2279 patients (1092 in AMT, 1027 in non-AMT, and 160 in pre-AMT groups). Higher rates of early discharge (without significant differences in the readmission rates) and shorter LOS were noted for the AMT patients. They were also more likely to be admitted into a ward allocated to their discipline and had lower bill size compared to non AMT patients.

**Conclusions:**

The AMT intervention improved patient outcomes and resource utilization. This model was noted to be sustainable and provides a potential solution for hospitals’ ED boarders who face a gap in inpatient care during their crucial first few hours of admissions while waiting for an inpatient bed.

**Electronic supplementary material:**

The online version of this article (doi:10.1186/s12913-017-2491-x) contains supplementary material, which is available to authorized users.

## Background

Hospitals around the world face a rising trend of high bed occupancy rates (BORs) and increased bed wait times for patients presenting to the Emergency Department (ED) and requiring admission [1]. These patients may spend long periods in ED as “boarders”, a term used to describe patients marked for admission who need to wait for an available bed. A prolonged ED stay has been associated with suboptimal patient outcomes, including higher mortality rates, longer length of hospitalization, higher risk of acquiring infections, and delays in definitive care such as antibiotic administrations for infections [2–6]. Another issue is the tendency to admit boarders to the first available bed in the hospital, leading to patients admitted to non-designated wards for the admitting specialty. These “outliers” have been shown to have worse outcomes than patients admitted to the designated specialty wards [7]*.*

ED boarders are an issue of patient care and safety affecting a large proportion of health care institutions around the world [8–10]. Although many solutions, such as admitting patients to inpatient hallways or assigning inpatient teams to boarders, have been tried in different health care systems, none have been shown to significantly improve clinical outcomes for this group of patients [11–13]*.*

Singapore is not exempt from the issues of rising health care demands. A rapidly aging population, with multiple co-morbidities, requiring hospital admission coupled with slower growth of inpatient capacity has led to high hospital BORs and increasing issue of ED boarders. [14]. Between 2005 and 2014, national acute hospital inpatient bed capacity grew at 0.54% annually whereas the rate of admissions grew at 2.03% annually [15]. ED boarders have become increasingly common and effective solutions are required for optimal patient care.

The objective of this study is to report on the results of a health service implementation involving the introduction of an physician led medical team providing inpatient care to boarders in a virtual ward setting within ED. We hypothesized that this early care would achieve similar, if not better, outcomes as management in a conventional inpatient ward, despite the limitations of being a virtual ward in an ED setting.

## Methods

### Setting and intervention

Our institution is a 1200-bed tertiary academic hospital, with around 14,000 ED presentations per month. National Standards for Healthcare requires patients to be admitted within 4 h of a decision to admit [16]. However, data from our institution (not included) showed that almost one third of patients requiring admission in 2013 had to wait for more than four hours with about 6% patients waiting up to 10 h for an inpatient bed. Additionally, 60% of general medicine (GM) patients resided outside the designated wards, with 25% being in surgical wards.

At our institution, inpatient care for hospitalized patients commences when a patient physically arrives at the assigned ward. This includes assessment, further investigation and initiation of treatment by an inpatient team, including physicians, nurses, allied healthcare staff and social workers. Such care is delayed when no inpatient bed is available and patient remains a “boarder” in ED. Boarders were conventionally cared for by ED team comprising of emergency specialists, trainees, and nurses. This posed an additional workload for ED team as their primary role is to triage and stabilize patients for transfer to the wards, intensive care, or discharge.

We developed a new care delivery system in our institution where all GM ED boarders were managed by designated GM physician teams called the Acute Medical Team (AMT) in a virtual inpatient ward set up in ED. The establishment of virtual ward allowed patients to access inpatient services, permitted only after an admission in the local health care system. GM ED boarders were transferred to the AMT if no physical inpatient bed was available within 2 h of the decision to admit. Although still residing in ED, these patients under AMT care were regarded as admitted patients in the electronic health record system and were eligible for all inpatient services, as if they were in an actual physical inpatient ward. These patients were then either discharged from AMT after treatment or transferred to a designated physical inpatient ward for further treatment, when an appropriate bed became available.

The development of this model required a multidisciplinary approach and support from hospital administration. The medical team in AMT consisted of inpatient physicians including GM consultants, and senior and junior internal medicine residents. Nursing support was provided by ED nurses who underwent a refresher skills training course to deliver inpatient nursing care. Similar to inpatient care, these patients received the full spectrum of services if required, including laboratory and diagnostic imaging services, pharmacy, allied health, and social workers, while they were still in the virtual ward. The finance department assisted in creating billing models for these inpatient services provided.

The AMT model was introduced in 2 phases, starting with patients admitted to the GM service during office hours (8 am-5 pm on weekdays and 8 am-noon on weekends) in April 2013 (phase 1). The coverage was later extended to provide 24-h service for GM patients in September 2014 (phase 2). The capacity of the virtual ward was 20 patients at any given time, based on the expected number of daily GM admissions in our institution.

### Study design and data collection

We undertook the current study to analyze patient outcomes as a result of the AMT intervention using a retrospective quasi-experimental cohort design [17]. To correct for learning lags during implementation of the AMT intervention, and to account for resource constraints in data collection, we collected the data at 6 time points between March 2013 and January 2015 that included a pre-AMT group (March 2013), one month after phase 1 intervention (May 2013), 6 months later (December 2013), pre-phase 2 (August 2014), one month post phase 2 (October 2014), and 3 months later (January 2015). We controlled for observable biases by matching the patients in each group by age and gender. Further analyses, described below, showed no differences between each group according to primary diagnosis, co-morbidities, nutritional or functional status, indicating no observable systematic biases between the test groups. We calculated the required sample size of each group to achieve statistical power, by considering the known variances and number of predictors for the model. For the pre-AMT group, we collected data on every fourth patient admitted to the general medicine wards in March 2013. For the AMT group, we collected data on all patients requiring admission to the GM service from ED but with no inpatient bed available within 2 h. Bed availability was the inclusion factor and there were no additional inclusion or exclusion criteria for transfer of care to AMT. For the non-AMT control group, we matched general medicine ward patients who were not admitted through the AMT for the same time periods, using the criteria described above. Ex-post analysis confirmed that selection to the AMT group was random as we did not detect day-of-week or time-of-day effects. As this was a retrospective review, no patients were lost to follow-up since we have complete records of their inpatient stay.

The primary outcomes studied included length of hospital stay (LOS), rates of early discharges (within 24 h), and placement in appropriate wards. Secondary outcomes included 3-month unplanned readmission rates and resource utilization, with patient’s hospital bill serving as a surrogate marker.

Data obtained from the hospital’s electronic medical records included patient demographics (age and gender), clinical parameters including Diagnosis-Related Groups (DRG) [18], comorbidities as measured by the Charlson Comorbidity Index (CCI) [19], 3-Minute Nutritional Screening score (3-MinNS) [20], and Katz functional status (Katz) [21]. The inpatient wards were categorized into 3 groups: Tier 1 wards were designated GM wards, Tier 2 wards were designated medical wards for other specialties (non-GM), and Tier 3 wards were surgical wards. AMT LOS was measured as the time under AMT care, from the point of AMT admission to either discharge or transfer to an inpatient unit. This was the time period that patients would have spent as boarders prior to the AMT intervention. LOS was measured as the number of days the patient was admitted in the hospital, commencing from the time of decision to admit in ED. Patients’ bill size for hospital stay was extracted from the hospital’s administrative billing system.

The institutional review board (National Healthcare Group Domain Specific Review Board [NHG DSRB]) approved the study (NHG DSRB Reference No: 2014/00975), and exempted it from written informed consent.

### Analyses

All statistical analyses were performed using the Statistical Package for the Social Sciences version 23 [22]. Descriptive data for quantitative variables (demographic, clinical data, LOS and bill size) were presented as median and interquartile ranges (IQR) due to the highly skewed data while categorical variables (gender, early discharge, readmission, and ward placement) were presented as number of cases and percentages (n, %). Chi-squared (χ^2^) tests were performed to compare the AMT and control groups (pre-AMT and non-AMT) for the categorical data, while comparison of the continuous variables, between the AMT and control groups, was computed using a non-parametric Mann-Whitney U test in order to account for the highly skewed LOS data where some values were zero. Statistical significance is set at *p* < 0.05 throughout. A Bonferroni correction was applied to account for increases in Type 1 errors when testing for the statistical significance in multiple pairwise comparisons.

## Results

The total study sample comprised of 2279 patients (1092 in AMT, 1027 in non-AMT, and 160 in pre-AMT group). Descriptive statistics are reported in Appendix 1 to 3 (Additional file 1). There were no significant differences at *p* < .05 between the groups in terms of age, gender, DRG, CCI, 3-MinNS, and Katz (Table 1).

**Table 1:** Demographics and clinical profiles of AMT and control-group patients

	Pre-AMT (*n* = 160)	AMT (*n* = 1092)	Non-AMT (*n* = 1027)	Comparison between AMT and Pre-AMT *p*-value	Comparison between AMT and non-AMT *p*-value
Age, median (IQR)	76 (61, 83.75)	74 (58, 83)	74 (57, 83)	*p* = 0.15	*p* = 0.78
Male, n (%)	71 (44.38)	493 (45.15)	425 (41.38)	*p* = 0.86^a^	*p* = 0.08^a^
CCI, median (IQR)	4 (3, 6)	4 (2, 6)	4 (2, 6)	*p* = 0.40	*p* = 0.96
DRG, median (IQR)	3 (3, 4)	3 (3, 4)	3 (2, 4)	*p* = 0.73	*p* = 0.47
3-MinNS, median (IQR)	0 (0, 2)	0 (0, 2)	0 (0, 2)	*p* = 0.30	*p* = 0.96
Katz, median (IQR)	4 (1, 6)	3 (1, 6)	2 (1, 6)	*p* = 0.51	*p* = 0.25

IQR = interquartile range of values between the 25th and 75th percentile

^a^ = comparison using x^2^-test

**Fig. 1:**
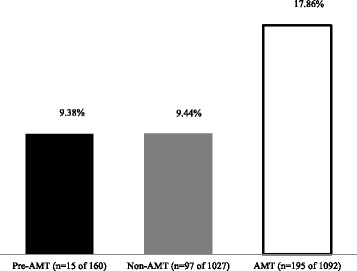
Comparison of early discharges between AMT and control groups

**Fig. 2:**
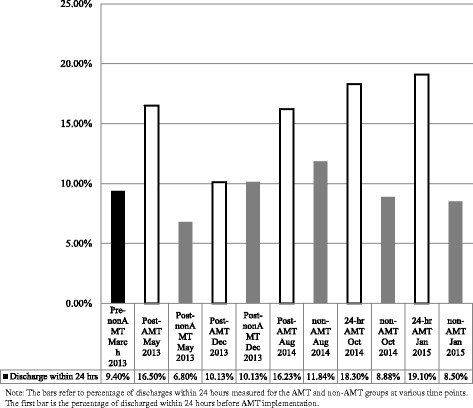
Percentage of early discharges between study groups at all time points

More AMT patients were discharged early (within 24 h) compared to other groups (AMT 17.86% vs pre-AMT 9.38%, *p* < .01 and non-AMT 9.44%, *p* < .01) (Fig. 1). Higher rates of earlier discharge from AMT were sustained at all study time points (Fig. 2). Although a larger proportion of patients in the AMT group were discharged within 24 h, the 3-month readmission rates of patients were not significantly different between the AMT and non-AMT groups (*p* = 0.23) (Table 2).

**Table 2:** LOS, Ward Placement, Unplanned Readmissions, and Bill Size Comparisons between AMT and control-group patients

	Pre-AMT (*n* = 160)	AMT (*n* = 1092)	Non-AMT (*n* = 1027)	Difference between AMT and Pre-AMT *p*-value	Difference between AMT and non-AMT *p*-value
Early Discharges, n (%)	15 (9.38)	195 (17.86)	97 (9.44)	*p* = 0.01	*p* < 0.001*
3-month Re-admission of Early Dischargers, n (%)	0 (0)	29 (15.8)	11 (11.34)	*p* < 0.001*	*p* = 0.23
3-month Unplanned Re-admissions, n (%)	43 (26.88)	252 (23.08)	247 (24.05)	*p* = 0.29	*p* = 0.60
Placement in Tier 1 Wards, n (%)	68 (42.5)	611 (55.95)	443 (43.14)	*p* < 0.001*	*p* < 0.001*
Placement in Tier 2 Wards, n (%)	54 (33.75)	226 (20.70)	375 (36.51)	*p* < 0.001*	*p* < 0.001*
Placement in Tier 3 Wards, n (%)	38 (23.75)	60 (5.49)	209 (20.35)	*p* < 0.001*	*p* < 0.001*
LOS in General Ward (days), mean (95% CI); median (IQR)	4 (2, 6)	3 (1, 7)	4 (2, 7)	*p* = 0.15^a^	*p* < 0.001^a^*
Bill size (S$) median (IQR)	2838.23 (1607.58, 5162.5)	2762.62 (1179.24, 5470.60)	3087.82 (1590.82, 5757.32)	*p* = 0.38^a^	*p* < 0.001^a^*

We applied a Bonferroni correction to compensate for Type 1 error in multiple pairwise comparisons. To maintain an overall α = 0.05 to reject, the Bonferroni correction for each individual hypothesis is α = 0.00625. Base on this, our test statistic is significant for 3-month readmission of early discharges, placement in tier 1,2 and 3 wards (AMT v Pre-AMT); early discharges, placement in tier 1, 2 and 3 wards, and bill size (AMT v non-AMT)

^a^comparison of original values using Mann Whitney U test

*significant after Bonferroni correction

The AMT LOS, the time patients were under AMT care rose from an average of 7 h in phase 1 to 16 h in phase 2, when AMT coverage became 24 h per day, reflecting the time inpatient care was brought forward. This was associated with an even higher percentage of early discharges. On average, 13.32% of patients were discharged after AMT care during phase 1 compared to 18.70% during phase 2 of the 24-h AMT coverage (Fig. 2).

**Fig. 3:**
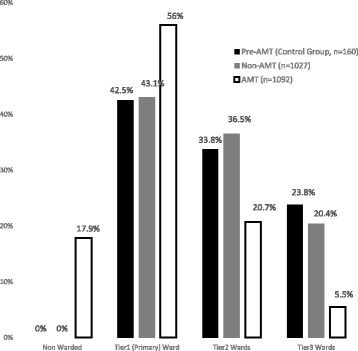
Inpatient bed placement

With AMT care, the pressure to move boarders quickly was eased and placement of patients in appropriate inpatient wards improved. Of all the patients admitted to a physical inpatient ward after AMT care, a significantly higher proportion was placed in appropriate wards. 56% of AMT patients were admitted to Tier 1 wards compared to 42.5% of pre-AMT and 43.1% of non-AMT patients (*p* < .01) (Fig. 3). Fewer AMT patients were placed in Tier 2 or 3 wards (26.2% AMT vs 57.6% pre-AMT and 56.9% non-AMT), reducing the scatter of GM patients outside of designated wards (*p* < .01) (Fig. 3).

Early specialist care and better inpatient placement improved efficiency, translating to a shorter median LOS of 3 days for AMT patients compared to 4 days for the other groups, despite no significant differences in DRG and CCI profiles. The average bill size was also lower for AMT patients compared to the non-AMT group (*p* < 0.001), largely driven by the shorter LOS. Table 2 summarizes the comparison of outcomes between the study groups for LOS, ward placement and bill size. Hierarchical logistic and multinomial regression analyses were performed to assess the incremental impact from AMT on early discharge and inpatient placement, respectively, after accounting for time period of the study and patients‘presenting conditions. The results in the Appendix 4 and 5 (Additional file 2) suggest that the AMT had a significant impact on early discharge and inpatient placement at *p* < .05.

Although data about ED operational metrics was not formally collected in this study, ED consult wait times (door to doctor time) remained stable throughtout study period, despite increased ED presentations.

## Discussion

We have shown that early specialist inpatient care by AMT in a virtual inpatient ward in ED may improve patient outcomes, which we defined as shorter LOS, a higher rate of early discharges with no rise in readmission rates, and better ward placement. Historically, these patients would have been waiting in ED for a bed to be available before review by specialists. The AMT model of care allows rapid assessment, investigations, and treatment within the critical first few hours of admission. This early definitive care resulted in a shortening of LOS for a large majority of patients with no adverse effects.

Our model has similarities to the acute medical or assessment units which have been successfully employed in some health care settings [23, 24]. However, there are notable differences. Our model involved a dedicated team of physicians who provided a full spectrum of inpatient care within ED. Compared to other acute medical unit models, there was no physical ward requiring accompanying resources, only modifications in administrative processes. Our unique hybrid care model involved inpatient physicians and ED nurses working alongside as one team to look after patients under AMT care.

Our care model is also different from the hospitalist system which involves admission to a physical bed and continuation of care by the same physician throughout the hospital stay [25]. Clinical teams in our healthcare system function around geographic locations, precluding this system. Another model that has been described in the literature includes transfer of patients to inpatient hallways during periods of high ED occupancy [13]. Patient outcomes in this study were similar to other patients admitted to general wards and no safety concerns were noted. This study also indirectly supports our hypothesis that improved patient outcomes are dependent on early specialist care and are not entirely related to the physical location of the patient.

An approach of assigning ED boarders to inpatient medical teams has not proven to be successful in another study. Patients still experience poorer outcomes as boarding time increased [6]. Prior to our intervention in Singapore, non-emergency inpatient investigations and consultations from other disciplines including allied health and medications could only be delivered if the patient was tagged to an inpatient location. Hence, assigning boarders to inpatient medical teams in our institution was felt to provide minimal benefit to patients. By establishing a dedicated medical team and virtual ward, these obstacles to early inpatient care were removed.

The AMT provided ED boarders with inpatient care while they waited for a bed in an inpatient ward. This eased the pressure to move patients to any available inpatient bed, facilitating more appropriate bed allocation for patients. Consequently, the number of patients admitted to Tier 2 and 3 wards fell as most patients were admitted to Tier 1 wards. This outcome facilitated right siting of inpatient care for GM (AMT) and freed up beds that GM patients would otherwise occupy in non-GM and surgical wards.

Early discharges and shorter LOS associated with AMT care also led to thousands of bed days saved for the hospital. AMT cared for 11,182 patients during the study period, and directly discharged 2795 patients without transfer to a physical inpatient unit; equivalent to 93 admissions avoided per month. Combined with a one day shorter LOS, the data suggests a savings of 16,772 bed-days during the study period due to the AMT care delivery model.

There are several potential reasons that this care model is able to discharge more patients home and avoid admissions. Firstly, inpatient care that is brought forward to the AMT patients while they waited in ED for a bed may have resulted in improved clinical conditions, leading to more direct discharges. Secondly, AMT patients were able to obtain outpatient management and early post-discharge follow-up care by the AMT GM physicians as needed, providing a safety net for supported discharges. Finally, the AMT delivered inpatient care with all the necessary consultations with subspecialty and allied health colleagues to provide comprehensive care and plan to support early discharges.

The principal limitation of this study was the inability to definitively specify cause-and-effect relationships between the reorganization of an admissions process and the improvement in clinical outcomes. For example, many readmissions to the hospital may not be preventable due to an aging population with chronic illnesses and high comorbidities. However, the use of the pre- and post-AMT control groups serve to mitigate questions of causality. As a real world study, we had to use a quasi-experimental cohort design to interpret the data. Randomization was not practical, but analyses of each time point showed similar improvements in outcomes from the AMT samples. Another limitation is that we did not collect data on ED operations, and so cannot comment definitively if the AMT positively or negatively impacted ED operations. In hindsight, this would have been valuable information, as we would have been able to report on the entire chain of care. A final limitation is related to external validity as the study was conducted in only one hospital. All these limitations represent opportunities for future research on this AMT model of care.

## Conclusions

The results of this study show that the AMT intervention, a virtual inpatient ward in ED, has the potential to improve patient outcomes and resource utilization. As acute hospitals ponder on ways to provide early care in the period between initial assessments in the ED and when inpatient care is available in the ward, a specialist Acute Medical Team that moves inpatient care “upstream” to where the patient is lodged in ED offers a potential solution. Thus, this model can bridge a gap in the care of ED boarders during the most vulnerable initial period of the admission.

## Additional files

Additional file 1: Appendix 1–3. Descriptive statistics of Pre-AMT, AMT, and Non-AMT enrolled patients. Appendices 1, 2, and 3 report the descriptive statistics for age, Charlson Co-morbidity Index, number of primary DRG codes, 3-MinNS, Katz Functional Score, Length of Stay, and bill size of Pre-AMT, AMT, and Non-AMT patients in the study period (March 2013 to January 2015). (DOCX 17 kb)

Additional file 2: Appendix 4–5. Regressions of AMT enrollment as a predictor of early discharge and inpatient bed placement. Appendix 4 reports on the logistic regression of AMT enrollment status on early discharge, after controlling for time of enrollment, age, gender, DRG, CCI, 3-MinNS, and Katz Score. The change in R2 reports on the statistically significant additional variance explained (18%) by AMT enrollment, after accounting for the effects of the control variables. The results indicate faster discharge for AMT patients, relative to pre-AMT and non-AMT patients. Appendix 5 reports on the multinomial regression of AMT enrollment status on inpatient bed placement, after controlling for time of enrollment, age, gender, DRG, CCI, 3-MinNS, and Katz Score. The change in R2 reports on the statistically significant additional variance explained (more than double) by AMT enrollment, after accounting for the effects of the control variables. The results indicate improved bed placement for AMT patients, relative to pre-AMT and non-AMT patients. (DOCX 18 kb)

## Abbreviations

3-MinNS: 3-Minute nutritional screening score
AMT: Acute Medical Team
BOR: Bed occupancy rate
CCI: Charlson Comorbidity Index (CCI)
CI: Confidence Interval
DRG: Diagnosis-Related Groups
ED: Emergency department
GM: General medicine
Katz: Katz functional status
LOS: Length of hospital stay
NHG DSRB: National Healthcare Group Domain Specific Review Board
