# Supplementary material for: Impact of inpatient Care in Emergency Department on outcomes: a quasi-experimental cohort study
Source: BMC Health Serv Res. 2017 Aug 14;17:555. doi: 10.1186/s12913-017-2491-x (PMC5557060; doi:10.1186/s12913-017-2491-x)
Supplement: Supplementary file 1 — Descriptive statistics of Pre-AMT, AMT, and Non-AMT enrolled patients. Appendices 1, 2, and 3 report the descriptive statistics for age, Charlson Co-morbidity Index, number of primary DRG codes, 3-MinNS, Katz Functional Score, Length of Stay, and bill size of Pre-AMT, AMT, and Non-AMT patients in the study period (March 2013 to January 2015). (DOCX 17 kb) [file 12913_2017_2491_MOESM1_ESM.docx]

**Appendix 1: Descriptive Statistics of Pre-AMT Data**

|  | Median (IQR) | Mean ± s.d. | Skewness ± s.e. | Kurtosis ± s.e. |
| --- | --- | --- | --- | --- |
| Age | 76 (61, 83.75) | 71.73 ± 17.14 | -0.72 ± 0.19 | -0.11 ± 0.38 |
| CCI | 4 (3, 6) | 4.27 ± 2.51 | 0.15 ± 6. 0.19 | 0.29 ± 0.38 |
| DRG | 3 (3, 4) | 3.06 ± 1.00 | -1.02 ± 0.19 | 0.04 ± 0.38 |
| 3-MinNS | 0 (0, 2) | 1.33 ± 1.91 | 1.68 ± 0.28 | 2.72 ± 0.56 |
| Katz | 4 (1, 6) | 3.43 ± 2.58 | -0.18 ± 0.22 | -1.77 ± 0.44 |
| LOS | 4 (2, 6) | 5.43 ± 6.24 | 3.51 ± 0.19 | 15.36 ± 0.38 |
| Bill Size S$ | 2838.23 (1607.58, 5162.52) | 4779.85 ± 8922.42 | 8.23 ± 0.19 | 83.17 ± 0.38 |

N = 160

**Appendix 2: Descriptive Statistics of AMT Data**

|  | Median (IQR) | Mean ± s.d. | Skewness ± s.e. | Kurtosis ± s.e. |
| --- | --- | --- | --- | --- |
| Age | 74 (58, 83) | 69.36 ± 17.93 | -0.81 ± 0.07 | -0.05 ± 0.15 |
| CCI | 4 (2, 6) | 4.07 ± 2.37 | 0.07 ± 0.07 | -0.25 ± 0.15 |
| DRG | 3 (3, 4) | 2.97 ± 1.12 | -0.83 ± 0.07 | -0.71 ± 0.15 |
| 3-MinNS | 0 (0, 2) | 1.01 ± 1.49 | 1.42 ± 0.19 | 1.14 ± 0.38 |
| Katz | 3 (1, 6) | 3.18 ± 2.46 | 0.04 ± 0.19 | -1.70 ± 0.38 |
| LOS | 3 (1, 7) | 5.83 ± 8.31 | 4.29 ± 0.07 | 29.63 ± 0.15 |
| Bill Size S$ | 2762.62 (1179.24, 5470.60) | 4633.42 ± 6211.83 | 4.35 ± 0.07 | 33.77 ± 0.15 |

N = 1092

**Appendix 3: Descriptive Statistics of Non-AMT Data**

|  | Median (IQR) | Mean ± s.d. | Skewness ± s.e. | Kurtosis ± s.e. |
| --- | --- | --- | --- | --- |
| Age | 74 (57, 83) | 69.09 ± 18.18 | -0.72 ± 0.08 | -0.27 ± 0.15 |
| CCI | 4 (2, 6) | 4.09 ± 2.51 | 0.18 ± 0.08 | -0.10 ± 0.15 |
| DRG | 3 (2, 4) | 2.93 ± 1.14 | -0.75 ± 0.08 | -0.89 ± 0.15 |
| 3-MinNS | 0 (0, 2) | 1.01 ± 1.55 | 1.63 ± 0.16 | 1.91 ± 0.33 |
| Katz | 2 (1, 6) | 2.86 ± 2.39 | 0.27 ± 0.16 | -1.52 ± 0.32 |
| LOS | 4 (2, 7) | 5.81 ± 7.49 | 5.58 ± 0.08 | 48.87 ± 0.15 |
| Bill Size S$ | 3087.82 (1590.82, 5757.32) | 5027.06 ± 6856.01 | 4.51 ± 0.08 | 26.68 ± 0.15 |

N = 1027
