# Supplementary material for: Impact of inpatient Care in Emergency Department on outcomes: a quasi-experimental cohort study
Source: BMC Health Serv Res. 2017 Aug 14;17:555. doi: 10.1186/s12913-017-2491-x (PMC5557060; doi:10.1186/s12913-017-2491-x)
Supplement: Supplementary file 2 — Regressions of AMT enrollment as a predictor of early discharge and inpatient bed placement. Appendix 4 reports on the logistic regression of AMT enrollment status on early discharge, after controlling for time of enrollment, age, gender, DRG, CCI, 3-MinNS, and Katz Score. The change in R2 reports on the statistically significant additional variance explained (18%) by AMT enrollment, after accounting for the effects of the control variables. The results indicate faster discharge for AMT patients, relative to pre-AMT and non-AMT patients. Appendix 5 reports on the multinomial regression of AMT enrollment status on inpatient bed placement, after controlling for time of enrollment, age, gender, DRG, CCI, 3-MinNS, and Katz Score. The change in R2 reports on the statistically significant additional variance explained (more than double) by AMT enrollment, after accounting for the effects of the control variables. The results indicate improved bed placement for AMT patients, relative to pre-AMT and non-AMT patients. (DOCX 18 kb) [file 12913_2017_2491_MOESM2_ESM.docx]

Appendix 4: Logistic Regression of AMT on Early Discharge

|  | Baseline on Early Discharge  (β [s.e.]) | Inclusion of AMT on Early Discharge  (β [s.e.]) |
| --- | --- | --- |
| Block 1: |  |  |
| May2013 | -.09 [.55] | -.53 [.60] |
| Dec2013 | -.10 [.50] | -.59 [.56] |
| Aug2014 | .24 [.63] | -.02 [.65] |
| Oct2014 | -.48 [.68] | -.94 [.73] |
| Jan2015 | .22 [.57] | -.07 [.59] |
| Age | -.03 [.02] | -.02 [.02] |
| Gender | .11 [.33] | .06 [.33] |
| DRG | -.32* [.14] | -.32* [.14] |
| CCI | .04 [.12] | .03 [.12] |
| 3-MinNS | -.02 [.13] | -.01 [.13] |
| Katz | .13 [.09] | .13 [.09] |
| Block 2: |  |  |
| **AMT Status** |  | .83* [.35] |
| Change in R^2^ |  | 2.4% |
| Adjusted R^2^ | 13.1% | 15.5% |

*p<.05, **p<.01, ***p<.001

Appendix 5: Multinomial Regression of AMT on Inpatient Bed Placement

|  | Baseline on Inpatient Placement  (β [s.e.]) | Inclusion of AMT on Inpatient Placement  (β [s.e.]) |
| --- | --- | --- |
| Block 1: |  |  |
| May2013 | -.20 [.54] | -.01 [.55] |
| Dec2013 | .44 [.43] | .74 [.46] |
| Aug2014 | .69 [.59] | .75 [.59] |
| Oct2014 | -.85 [.68] | .15 [.15] |
| Jan2015 | -.68 [.60] | -.60 [.60] |
| Age | .01 [.02] | .02 [.02] |
| Gender | -.14 [.34] | -.14 [.34] |
| DRG | .02 [.04] | .08 [.14] |
| CCI | .01 [.11] | .01 [.11] |
| 3-MinNS | .08 [.11] | .08 [.11] |
| Katz | .07 [.08] | .07 [.08] |
| Block 2: |  |  |
| **AMT Status** |  | -1.00* [.45] |
| Change in R^2^ |  | 17.7% |
| Adjusted R^2^ | 13.3% | 31.0% |

*p<.05, **p<.01, ***p<.001
